# Motivated and without Fear of Failure: The Strength of Basic Psychological Needs in Youth Spanish Athletes in Team Sports

**DOI:** 10.5114/jhk/162449

**Published:** 2023-04-20

**Authors:** Juan González-Hernández, Manuel Gómez-López, David Manzano-Sánchez, Alfonso Valero-Valenzuela

**Affiliations:** 1 Department of Personality, Evaluation and Psychological Treatment, University of Granada, Granada, Spain.; 2 Department of Physical Activity and Sport, University of Murcia, Murcia, Spain.; 3 Campus of International Excellence “Mare Nostrum”, University of Murcia, Murcia, Spain.; 4 Department of Didactics of Musical, Plastic and Corporal Expression, University of Extremadura, Badajoz, Spain.

**Keywords:** sport, emotional self-regulation, self-motivation, young athletes

## Abstract

Connecting desires for achievement, the satisfaction of basic psychological needs and the perception of fear of failure is one of the most relevant questions in the understanding of negative mental responses in youth athletes. How to act with less fear is what every athlete seeks to feel to enhance their performance actions. This paper aims to shed light on a sample of 681 members of sports teams belonging to different Spanish clubs (391 boys and 290 girls), with a mean age of 16.2 years, and a high sports dedication (75.5% > 5 years of experience; 96.3% > two training sessions/week; 90.3% > 3 hours of training/week). The collected data used self-reports based on the tenets of achievement motivation, Self-Determination Theory, and fear of failure. Those aspects linked to task involvement were positively close to Basic Psychological Needs (BPNs), while those related to ego involvement moved away from task involvement and BPNs. Fear was associated positively and significantly only with ego, and negatively with the rest of the constructs. In the standardized direct effect, positive and significant associations were observed among all constructs except between an ego-involving climate and basic psychological needs satisfaction. The association between a task-involving climate and BPNs was significant in fostering relationships among group members, as well as in improving interpersonal cohesion, empathic understanding processes, and reducing fear of failure in youth athletes.

## Introduction

For athletes, achieving success by being competitive is their ultimate goal. According to the literature on competitiveness, this requires clear achievement motivation, the reduction of failure avoidance attitudes (Martens, 1975; Reese et al., 2022), interpretation of well-being directly linked to pleasure, as well as perceptions of autonomy, competence and the climate perceived by the athlete in his or her psychosocial interaction with the sporting context (Moreno-Murcia et al., 2019; Passos et al., 2018).

From a multidimensional perspective, the motivation of an athlete to compete or seek success considers both the motives of personal achievement, which involve goals of improvement and/or sporting excellence, as well as the psychosocial climate created to achieve and facilitate their best performance. Thus, managing and controlling the sports situation will depend on both personal factors (e.g., self-esteem, rigid behavioural beliefs) and social factors (e.g., coaches' styles, social support) (Carvalho et al., 2018; Díaz-García et al., 2021; Gillet and Rosnet, 2008).

The model of Basic Psychological Needs (BPNs), as one of the sub-theories of Self-Determination Theory (SDT) (Deci and Ryan, 1985; Ryan and Deci, 2017), seeks to understand the human processes of motivation in various areas such as sport, describing the importance of competence (feeling competent and self-efficient in the performance and control of tasks and learning), autonomy (the athlete's need to feel the origin of their actions, taking responsibility for their actions and showing freedom of action) and social relations (connection with others) in the various contexts in which athletes develop and manifest themselves (García Calvo et al., 2012).

In the context of sport, when the satisfaction of BPNs is experienced in a specific situation, more intrinsically motivated sports behaviours emerge (e.g., effort, involvement, pride, identification) (Ryan and Deci, 2017), whereas the experience of feeling frustrated (or rather the frustration of these basic needs) results in the emergence of discomfort and adaptive maladjustments (e.g., anxiety, fear, burnout) (Brown et al., 2017). According to the continuum of self-determination theory, the more and better the positive perceptions of autonomy, competence, and social bonding are in athletes (satisfaction of basic needs), the greater will be their intrinsic motivation and their perception of control of actions and situations (inside and outside sport contexts), reducing those extrinsic (and less controllable) motivational aspects that also accompany sports behavior.

**Figure 1:**
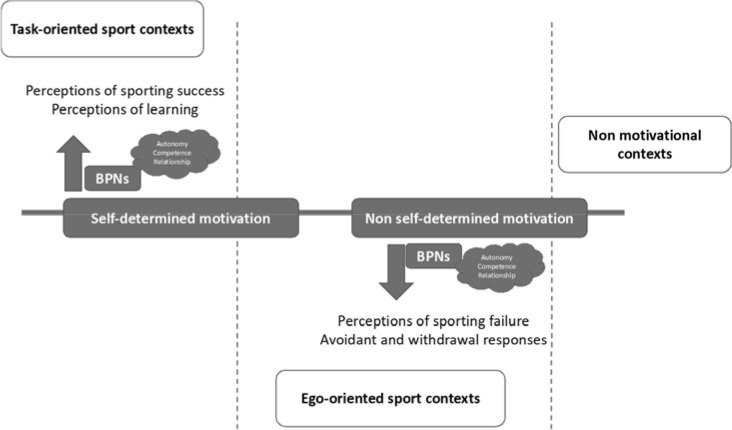
Motivational processes and tendencies towards perceived failure/success.

Perceiving the moment of failure is primarily connected to feelings of competence, and when this occurs in a social context observed by others where there are socio-emotional ties (e.g., expectations, promises, commitments), it exacerbates and infects perceptions of being autonomous and not dependent on anyone (Figure 1). A more self-determined disposition ensured through the satisfaction of BPNs could decrease athletes' negative feelings of stress, tension, anxiety, and avoidance behaviour regarding fear of making a mistake (Ruiz-Sánchez et al., 2017; Wikman et al., 2014).

### The Relevance of the Motivational Context and the Young Athlete's Fear of Failure

The contextual influence of sport on the young athlete (mainly from coaches, parents and peers) has not been sufficiently considered in the scientific literature. From the contextual approach to achievement motivation (Nichols, 1989), it is argued that those elements of the social environment that orient the athlete towards the tasks to be performed, enhance task-focused motivation, and consequently predict self- determined motivation (Reinboth and Duda, 2004). On the other hand, those environmental forces that build an ego-oriented motivational climate, emphasize interpersonal comparison and intra-group competition, triggering negative motivational outcomes (e.g., distress, reduced effort), among others (O'Rourke et al., 2014).

Estimation of the consequences of failure provokes defensive reactions in athletes (e.g., stress, worries) with different consequences (e.g., behavioural inhibition, fears, suffering) (Wikman et al., 2014). In this sense, in addition to age or experience gained (Sagar and Lavallee, 2010; Sagar et al., 2012), fear of making a mistake becomes a variable of interest to mediate those contextually created motivational processes and the effective response necessary for adequate sports performance (Correia et al., 2017; Taylor et al., 2021). The strength of social valuation (e.g., technical feedback received, social approval from peers) is the main source of concern for athletes (Reinboth and Duda, 2004). Its relationships with self-esteem and the difficulty of forming self-determined motivation, make the occurrence of failure a source of social conflict (González-Hernández et al., 2022; Sagar et al., 2007) and abandonment of the action to be performed.

**Figure 2:**
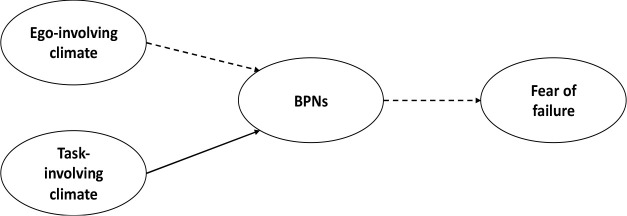
Hypothesized model.

Having reviewed the literature to check the fulfilment of the hypothesized model (Figure 2), the present work pursued the following objectives: a) to establish relationships between achievement contexts, the satisfaction of basic psychological needs and perceptions of fear of failure in a sample of young athletes, and b) to describe the mediating value of basic psychological needs between motivational contexts and the occurrence of fear of failure in youth athletes.

## Methods

### Participants

Descriptive analysis and sample distribution are presented in Table 1. A total of 681 team sports players (basketball and handball) belonging to different Spanish clubs participated in the study (391_boys_, 57.4%; 290_girls_, 42.6%), with an average age of 16.2 years (*SD* = 0.92). Most of them stated that they had more than 5 years of sports experience (75.5%; *n* = 514), and they trained more than twice/week (96.3%; *n* = 656) and more than 3 hours/week (90.3%; *n* = 615).

**Table 1:** Socio-demographic and sporting characteristics of participants.

		N	%
Gender	Boys	391	57.4%
	Girls	290	42.6%
Age-Game Category	Cadets (14–15 years old)	142	20.9%
	Youth (16–17 years old)	539	79.1%
Sports Experience	up to 5 years	167	24.5%
	more than 5 years	514	75.5%
Trainings/Week	Up to 2 training sessions/week	25	3.7%
	More than 2 training sessions/week	656	96.3%
Training Hours/Week	Up to 3 hours/week	66	9.7%
	More than 3 hours/week	615	90.3%

### Measures

*Performance Failure Appraisal Inventory* (PFAI; Conroy et al., 2002). The Spanish version (Moreno-Murcia and Conte, 2011) includes 25 items grouped in 5 first-order factors and 1 second-order factor. For the present study, the second-order factor was taken into account (e.g., *“When I am not succeeding, I am less valuable than when I succeed”*; “*When I am failing, my future seems uncertain”*). Responses were provided on a 5-point Likert scale ranging from 1 (do not believe at all) to 5 (believe 100% of the time).

*Perceived Motivational Climate in Sport Questionnaire* (PMCSQ-2; Walling et al., 1993). The Spanish version (Balaguer et al., 1997a, 1997b) includes 29 items grouped in two dimensions: ego-involving (competitive) climate (14 items, e.g., *“On this team, the coach gives most of his or her attention to the stars”*) and the task-involving (mastery) climate (15 items, e.g., *“On this team, the coach emphasizes always trying to do your best”*). Responses were provided on a 5-point Likert scale ranging from 1 (strongly disagree) to 5 (strongly agree).

*Basic Psychological Needs in Exercise Scale* (BPNES; Vlachopoulos and Michailidou, 2006). The Spanish version (Sánchez and Núñez, 2007) includes 12 items (e.g., *“The way I exercise is in agreement with my choices and interests”*; *“I feel I perform successfully the activities of my exercise programme”*; *“My relationships with the people I exercise with are close”*). Responses were provided on a 5-point Likert scale ranging from 1 (I don’t agree at all) to 5 (I completely agree).

### Design and Procedures

A letter explaining the objectives of the research and how it was to be carried out, accompanied by two models of informed consent and permission (one for the parents and another for youth athletes), together with a copy of the measures, was sent to the clubs (first to presidents) before the data collection. The questionnaire was administered by the researchers in the sports facilities of the clubs during training sessions and completed by participants in 20–30 min. All the participants were informed of the objectives and their rights as participants in the study, as well as of the voluntary nature and the absolute confidentiality of the answers and handling of the data. It was explained that there were no correct or incorrect answers, so that participants would answer honestly. This study was carried out following the ethical guidelines of the American Psychological Association (APA) and the Declaration of Helsinki (2013), and the protocol was approved by the Ethics Committee of the University of Murcia, Spain (ID: 1494/2017).

### Statistical Analysis

Means, standard deviation, Cronbach reliability with alpha > 0.70 values were considered acceptable for the psychological mastery scales (Nunnally, 1978), and bivariate correlations were analyzed for all variables under analysis. A two-step maximum likelihood (ML) approach suggested by Kline (2016) in AMOS 23.0 (SPSS Inc., Chicago, IL, USA) was performed. Firstly, confirmatory factor analysis (CFA) was performed to analyze the psychometric properties of the purposed model. Discriminant validity was established when the correlation coefficients were lower than average variance extracted (AVE) for each construct exceeding the squared correlations between that construct and any other construct (Fornell and Larcker, 1981; Hair et al., 2014). Secondly, a structural equation model (SEM) was performed to test proposed relationships among different constructs. The following were considered: the Comparative Fit Index (CFI), Normalized Fit Index (NFI), Standard Root Mean Residual (SRMR), and Root Mean Square Error of Approximation (RMSEA) with its Confidence Interval (CI: 90%). For these indices, scores of the CFI and NFI > 0.90 SRMR and RMSEA < 0.08 were considered acceptable, following several recommendations (e.g., Byrne, 2010; Marsh et al., 2004).

### Mediation Analysis

For mediation analysis, the direct and indirect effects among constructs on outcome variables were analyzed as suggested by Hair et al. (2014) and Hayes (2018). To analyze the significance of direct and indirect effects, the bootstrap resampling procedure (1000 samples) via AMOS 24.0 was performed through corrected confidence intervals (CI:95%). The indirect effect was considered significant (< 0.05) when its confidence interval did not include zero, as suggested by several authors (e.g., Hayes, 2018; Williams and MacKinnon, 2008).

## Results

### Preliminary Analyses

First, we performed CFAs to test the factorial structure of each scale, exhibiting acceptable to good fit indices with significant factor loadings (except the PMCSQ-2). Due to CFA for the PMCSQ-2 showed unacceptable fit indices, it was decided to exclude those items with factor loading less than 0.50 as suggested by Kline (2016). As a result, the PMCSQ-2 was composed of seven items in the task factor and six items of the ego factor.

### Measurement Model

Table 2 shows the bivariate correlations among variables. The task involving construct was positively and significantly associated with BPNs, whereas ego was negatively and significantly associated with the task factor involving BPNs. Fear was associated positively and significantly only with the ego-involving climate, nd negatively with the rest of the constructs. All constructs present adjusted values of composite reliability, all greater than 0.70 (Hair et al., 2014).

**Table 2:** Bivariate correlations among variables.

Constructs	α	1	2	3	4
Task involving (1)	0.85	-	-	-	-
Ego involving (2)	0.84	−0.525**	-	-	-
BPNs (3)	0.71	0.416**	−0.164**	-	-
Fear of failure (4)	0.70	−0.270**	0.491**	−0.234**	-
CR		0.814	0.821	0.759	0.908

Note. BPNs = basic psychological needs (composite factor); SD = standard deviation; CR = composite reliability; ** p < 0.01

The measurement model test included task- and ego-involving climates, basic psychological needs satisfaction, and fear of failure. Results showed a good fit of the data (chi2 = 552.0 (176); SRMR = 0.051; RMSEA = 0.056 [90% CI = 0.051, 0.061]; TFI = 0.939; CFI = 0.939). Additionally, the measurement model revealed no problems with convergent and discriminant validity, since the average variance extracted (AVE) was greater than or equal to 0.50 (except for task and ego involving climate dimensions) (Hair et al., 2014; Forner and Larcker, 1981), and the square correlations among all constructs were less than the AVE of each factor (Forner and Larcker, 1981).

### Structural Model

**Figure 3:**
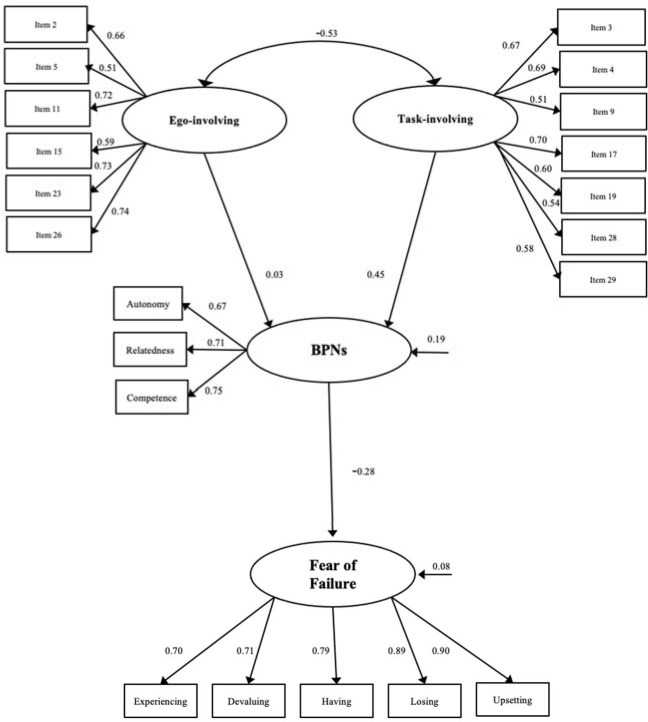
Standardized individual variables. Hypothesized model.

The structural model demonstrated a good fit of the data (chi2 = 549.6 (176); chi2/df = 3.12; SRMR = 0.102; RMSEA = 0.056 [90% CI = 0.051, 0.061]; TLI = 0.927; CFI = 0.939). In the standardized direct effect (Figure 3), positive and significant associations were observed among all constructs, except between an ego-involving climate and basic psychological needs satisfaction. Specifically, the associations between a task-involving climate and basic psychological needs (β = 0.45), as well as basic psychological needs and fear of failure (β = −0.28) were significant. The association between an ego-involving climate and basic psychological needs was not significant (β = 0.03).

## Discussion

To expand the athlete-sport context interaction, the present study aimed to determine the relationships between achievement contexts, the satisfaction of basic psychological needs, and the perception of fear of failure in a sample of youth athletes, as well as describe the mediating value of basic psychological needs between motivational contexts and the occurrence of fear of failure in youth athletes.

Those athletes who perceived themselves to be in contexts towards greater task involvement showed positive directionality with BPN, whereas those whose perceptions were focused on ego involvement showed negative links between task involvement and BPN. Young athletes learn best when the climate is cooperative (Weiss et al., 2021), offering the opportunity to perceive the involvement and importance that each team member offers, together with the orientation towards effort and the positive value of individual improvement, prioritization of effort and self- improvement (Remor, 2007). In this sense, authors like Amado et al. (2019) concluded that team sports coaches should promote a perception of autonomy and competence in their athletes, with the aim of enhancing more positive self-talk and self-motivational processes in competition, which promote better personal and team performance and well-being.

Iglesias et al. (2020) pointed out that teammates who help each other to progress, together with the union and teamwork enhanced by the coach's message, produce an increase in autonomy, feel more competent by knowing their roles and focusing on their function, and maintain much more enriched social relationships. Li et al. (2019), in a study with 271 different athletes, found that a climate built around the tasks set by the coach and teammates, enhanced the feeling of competence and self-improvement as a goal, positively affecting athletes’ mental strength.

Fear of failure responses showed positive relationships when the motivational perception of the context was ego-involvement oriented, whereas they were negative when the orientation was task-oriented and with the satisfaction of basic psychological needs. Enhancing the capacities for self-control, autonomy and competence reduces individual and collective fear of failure. Furthermore, connectivity among team members is enhanced by social understanding processes (e.g., empathy), hence BPNs predict and mediate between a task-oriented climate and the reduction of fear of failure (Sagar and Jowett, 2015). The development of quality relationships characterized by affective closeness, commitment, complementary transactions, empathy, as well as the possession of self-control, are key factors in reducing fear of failure among individuals (Taylor et al., 2021). The literature has demonstrated the usefulness of BPNs as a protective factor against the fear of failure in studies carried out in sports (Seker, 2019) and educational contexts (Gómez-López et al., 2021). In a study developed by Gómez-López et al. (2021), the satisfaction of BPNs of 464 secondary education students and their relationship with the aversive causes of fear of failure in physical education (PE) classes were analyzed. The results showed that the perception of autonomy was related to a lower fear of losing the interest of others, while the perception of competence together with the relationship with others were related to all aversive causes of fear of failure. The results showed that BPN satisfaction in PE classes was negatively related to aversive behaviors of fear of failure in the development of the activities proposed by the teacher.

As expected, the hypothesized model holds for the predictive relationships between the perceived motivational climate and basic psychological needs. Both Achievement Motivation Theory and Self-Determination Theory have consolidated a significant body of argumentation on the relevance of ego-orientation to frustration and task-orientation to the satisfaction of BPNs. This study reaffirms this information and shows how BPNs reduce the appearance of fear of failure responses.

The present study has some limitations that need to be addressed. Although the sample size is considerable, its cross-sectional design limits the generalization of the results to different aspects of sporting activities (e.g., matches, training, end of the season, last years in the category, years together playing with the same teammates) and the specificity of the sample analyzed. Thus, future replication in different playing categories is required (e.g., youth, juniors, U23), supported by instruments that are more closely matched to the study sample (Monteiro et al., 2018) or under longitudinal designs that facilitate checking the evolution of both the contextual influence and the perception of BPN satisfaction and fear of failure. In addition, the model presented is one of the valid options among the hypothetical ones that may exist. Other models that include each of the basic psychological needs separately could also be valid, and the relationships and values presented in this current model could be modified. In addition, each of the separate BPNs could be influenced to different extents by the motivational climate and fear of failure.

## Conclusions

According to the hypothesized and contrasted model, it can be extracted from the present work that the construction of motivational climates focused on tasks and responsibilities of action enhances BPNs and reduces fear of failure responses. There is evidence of the relevance of some ego-focus in predicting the satisfaction of the needs for social relations, autonomy, and competence, with a positive impact on lower levels of fear of failure.

With an applied orientation, and considering the view of coaches, it is important to build contexts that encourage the roles, responsibilities, and details that sports actions offer to the athlete, giving them the opportunity to perceive themselves with greater autonomy and, above all, with greater competence at the time of their execution. Moreover, fostering relationships between group members improves interpersonal cohesion, cooperativity, and empathic understanding processes. This will allow not only the transfer of responsibility to each athlete in common tasks, but also the understanding of the tasks of others, their limitations, and the potential to observe a balanced context where the protagonism is shared. Together with this, enhancing their autonomy and perception of competence will allow to gain the necessary security to reduce the fear of failing and the embarrassment of others seeing them fail.

Other strategies to improve the satisfaction of individual needs for competition and autonomy are aimed at rewarding effort instead of results, encouraging the evaluation of the young athlete towards intrapersonal indices associated with responsibility and the feeling of freedom, or sharing and committing to proposed and attainable objectives, which will favour decision-making and daring in training and, above all, in competitions.
